# Interscalene and Erector Spinae Block Combination to Treat Latissimus Dorsi Repair: A Case Report

**DOI:** 10.7759/cureus.45424

**Published:** 2023-09-17

**Authors:** Ashley H Yi, Edwin C Lin, Paul S Lee

**Affiliations:** 1 Anesthesiology, University of Southern California, Los Angeles, USA; 2 Regional Anesthesiology, Hospital for Special Surgery, New York, USA

**Keywords:** latissimus dorsi rupture, regional anesthesiology, postoperative pain relief, interscalene nerve block, erector spinae plane block

## Abstract

Latissimus dorsi (LD) tendon rupture is a rare injury that occurs in athletes throughout a wide range of sports, including baseball, rock climbing, tennis, and golf. LD tendon repair requires analgesia in nerve distributions from C5-T6. A 33-year-old man presented for right LD tendon repair after rock climbing eight weeks prior to the operation. An interscalene nerve block catheter was placed preoperatively for postoperative pain control. After induction of general endotracheal anesthesia, a mid-axillary incision was made down to the sixth rib and the patient underwent LD tendon repair. Postoperatively, the patient reported decreased pinprick sensation at the shoulder but pain along the mid-axillary incision. The erector spinae plane block was placed at the T3 level and pain relief was achieved within 20 minutes. This case report demonstrates that the erector spinae plane block serves as a useful adjunct to the brachial plexus block in surgeries involving the LD tendon.

## Introduction

Rupture of the latissimus dorsi tendon is a rare injury more often occurring in professional sports such as baseball, rock climbing, tennis, and golf. When surgical repair is recommended, a mid-axillary to posterior axillary incision is commonly made during the procedure for tendon retrieval [1]. Thus, patients undergoing surgical repair require analgesia in a widespread distribution from C5-T6 to adequately cover muscle pain and postoperative incisional pain across several thoracic dermatomes. Interscalene nerve blocks are routinely performed on patients undergoing shoulder, upper arm, or elbow surgery to provide reliable anesthesia to most of the brachial plexus, typically C5-C7 [2]. However, these interscalene nerve blocks fail to address incisional pain caused by surgeries involving the LD tendon due to the thoracic dermatomes in this area. Alternatively, erector spinae plane blocks deliver multi-dermatomal analgesia in the anterior, posterior, and lateral thoracic and abdominal areas and serve as an effective adjunct [3].

In this case report, we present a surgical repair of a rare LD tendon rupture that required a combination of interscalene and erector spinae plane (ESP) nerve blocks to achieve adequate pain control.

## Case presentation

A 33-year-old man (180 cm, 73 kg) suffered a full-thickness LD tendon tear while rock climbing and was scheduled for a right LD tendon repair eight weeks after his injury. His past medical and surgical history was significant only for mild asthma and a previous pectoralis major tendon rupture that was repaired in 2011. He was not taking any medications.

An interscalene nerve catheter was placed in the preoperative holding area for postoperative pain control. A 20 cc solution of 0.5% ropivacaine was injected via an 18-gauge 100 mm echogenic needle with ultrasound guidance. A 20-gauge catheter was then threaded into the needle and appropriately dressed on the skin. Aspiration was negative and no paresthesia was experienced.

The patient was administered general endotracheal anesthesia with 60 mg lidocaine, 180 mg propofol, and 60 mg rocuronium. For analgesia, the patient received 50 mcg of fentanyl, 30 mg of ketamine, 10 mg of dexamethasone, and 30 mg of ketorolac. Standard ASA monitoring and noninvasive blood pressure monitoring were maintained. The surgeon performed an incision starting along the mid-axillary line down to the sixth rib and dissected inferiorly along the chest wall until the thoracodorsal nerve was identified and tracked to locate the LD muscle. Proximal dissection along the muscle identified the LD tendon stump, which had ruptured alongside a significant portion of the teres major and demonstrated scar formation. A small portion of the tendon was atrophied but the LD muscle belly appeared healthy and fired with checkpoint nerve stimulation. Continuation of the proximal dissection revealed the humeral shaft and medial aspect of the biceps tendon. Suture anchors were placed on the humerus to recreate the footprint of the LD tendon. The LD tendon was reduced down to its insertion on the humerus using Krackow stitches and a few Mason-Allen throws. After successful LD tendon repair, the patient recovered and his interscalene catheter was connected to an elastomeric local anesthetic pump full of 0.2% ropivacaine. He was discharged home the same day with the interscalene catheter running at 4 cc/hr [4].

**Figure 1:**
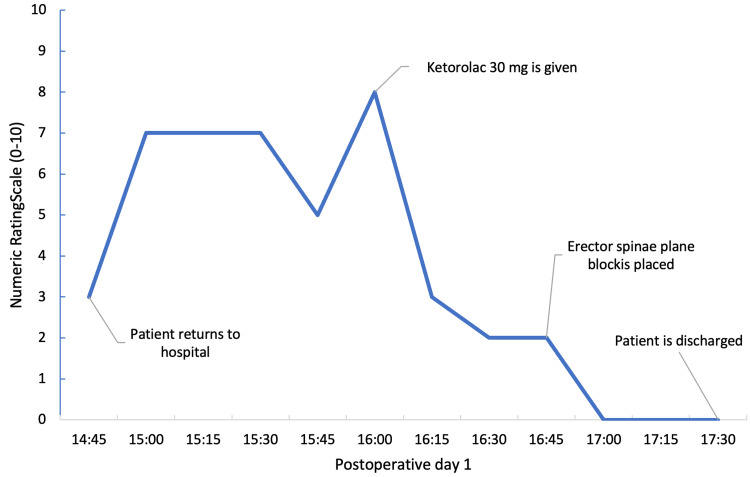
Progression of pain relief on postoperative day one

As per institutional protocol, the patient was called on postoperative day one to assess his pain. At the time of the phone call, the pain was a 1 out of 10 on the numerical rating scale (NRS). Later in the day, the patient called to report an NRS of 10 despite increasing the interscalene catheter rate to 12 cc/hr and taking hydrocodone/acetaminophen as prescribed. Due to the intense pain, the patient was prompted to return to the hospital for evaluation. On evaluation, the patient had decreased pinprick sensation at his shoulder and pain along his mid-axillary incision. The incision was healing well with no fluctuance, drainage, or erythema noted on exam. Ketorolac 30 mg was given for interim pain relief, and the nerve block was reassessed by the anesthesia team. Given that the interscalene nerve block did not adequately cover his pain, the plan was to supplement with an ESP single-shot block. After the patient was positioned prone, the right T3 and T4 transverse processes were identified under ultrasound guidance using a linear transducer (6-13 MHz; Fuji Sonosite, Bothell, WA). Once local anesthesia was injected into the skin, a 21-gauge 100 mm needle was inserted and advanced to the T3 transverse process. Upon bony contact, normal saline was used to confirm lifting of the erector spinae muscle whereupon 30 cc of 0.25% bupivacaine with 10 mg of dexamethasone was injected. The patient reported pain relief within 20 minutes of the procedure (Figure 1). The interscalene catheter rate remained at 8 cc/hr. On postoperative day two, the patient reported significant improvement in pain and we removed the catheter without issue on postoperative day three. His pain continued to be well-controlled on oral pain medications.

## Discussion

The latissimus dorsi muscle is a broad back muscle that arises from the inferior thoracic and lumbar spinous processes, iliac crests, and lower ribs to insert into the intertubercular groove of the humerus. LD tendon injuries rarely occur in isolation, often involving the teres major tendon or other adjacent shoulder girdle structures. LD tendon rupture repair requires analgesia in the brachial plexus as well as the following affected regions: C5-C8: Lower subscapular nerve and thoracodorsal nerve to teres major; C6-C8: Thoracodorsal nerve to latissimus dorsi; C8-T1: Medial brachial cutaneous nerve - cutaneous axillary incision; T2: Intercostobrachial nerve - cutaneous axillary incision; T1-T6: Intercostal nerves - cutaneous axillary incision; Posterior proximal humerus: Long thoracic (C5-C7) and suprascapular (C5-C6); Posterior medial humerus: Radial nerve (C5-T1).

The originally placed interscalene block could be effective for partial coverage of muscle pain to the teres major, latissimus dorsi, and humerus. However, it did not cover pain from the mid-axillary incision that brought the patient back to the hospital.

Erector spinae plane blocks are fascial plane blocks utilized for sensory nerve blockade in the thoracic or lumbar levels depending on the level of placement. Case reports of ESP blocks have demonstrated them to be phrenic nerve-sparing [5,6], thus preserving the mobility of the arm and hand and diaphragmatic function. CT imaging of cadavers and live patients demonstrate variable but potentially extensive spread in the ESP and paravertebral space, with a median volume needed to cover one dermatome of 3.4 cc (ranges from 2.5 cc - 6.6 cc); a 30 cc bolus in this review reached a maximum of nine dermatomes [7]. A continuous ESP block has been reported in a case report with efficacy for analgesia for sternum closure utilizing a latissimus dorsi muscle flap [8].

**Figure 2:**
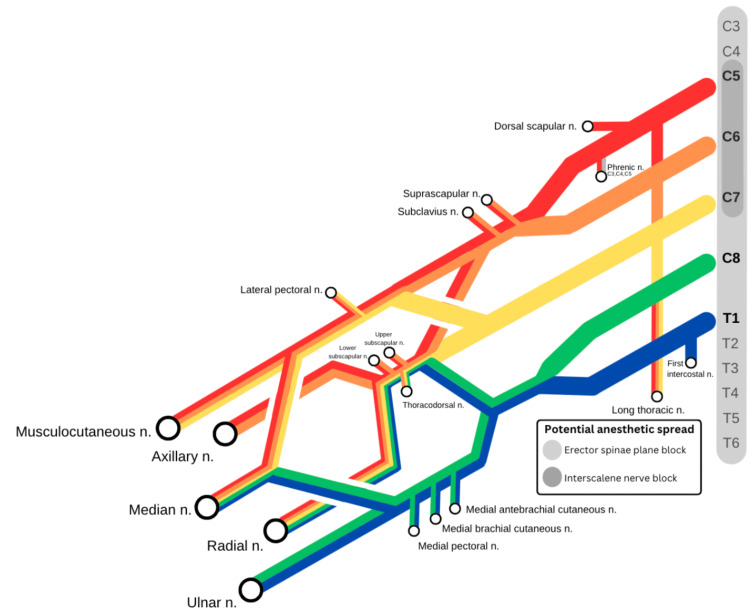
Brachial plexus and associated coverage by both interscalene and T3 erector spinae block Image created by Ashley H. Yi

This T3 ESP block placed for this patient may have covered the patient’s mid-axillary skin incision and part of the lower brachial plexus, assuming a conservative spread of 6 dermatomes (T1-T6) [7]. A case report of ESP block at T2-T3 in five patients undergoing shoulder surgery provided excellent analgesia, with a spread of local anesthetic demonstrated on CT with paravertebral spread reaching the C3-4 level with 20 cc of diluted contrast injected through the catheter [6]. Additionally, two case reports of ESP blocks at C7 have demonstrated sensory blocks from C3-T6 and C5-T5 [5]. Figure 2 illustrates the potential anesthetic spread by interscalene and ESP blocks.

Given the thoracic dermatomes affected by this surgery, there are other potential choices for regional blockade. One option is a paravertebral block, shown to be effective in previous literature for latissimus dorsi flap breast reconstruction [9]. Other truncal blocks that may be of benefit are pectoral (PECS) and serratus anterior plane blocks [10-13]. A thoracic epidural would undoubtedly be beneficial here as well, but given the outpatient nature of this surgery, it would not be as practical. A final option could be placing a single T1-T2 ESP block in hopes that it would cover both the cervical and thoracic dermatomes involved in this case. Given all these options, a discussion with the surgical team regarding the surgical approach may also be helpful in the appropriate selection of nerve blocks before proceeding.

Further studies are needed to determine the optimal analgesic management of surgeries involving the latissimus dorsi. Our case report, however, shows that the ESP block serves as a useful block in conjunction with an interscalene block.

## Conclusions

Latissimus dorsi tendon injuries are commonly treated nonoperatively; surgical repair is recommended only for professional athletes whose throwing ability depends on regaining LD function without relying on compensation by other muscles. The single-incision approach via a posterior axillary incision as taken in this case requires analgesia in a widespread distribution from C5-T6. This case report demonstrates that pairing an interscalene block and an ESP block for multi-dermatomal analgesia is effective in blocking the brachial plexus as well as addressing muscle and incisional pain. Given the rarity of surgical repair, there is no anesthetic standard of care for patients with latissimus dorsi tendon injuries, and this case report offers an effective method of achieving satisfactory pain control for these patients.
